# Urine sample for HPV detection in men: is it a valid and non-invasive diagnostic alternative?

**DOI:** 10.1128/spectrum.00440-26

**Published:** 2026-06-25

**Authors:** Arianna Sucato, Nicola Serra, Michela Buttà, Leonardo Di Gregorio, Manfredi Del Sordo, Daniela Pistoia, Alberto Firenze, Maurizio Soresi, Gabriele Tulone, Giuseppina Capra

**Affiliations:** 1 Department of Health Promotion, Mother and Child Care, Internal Medicine, Medical Specialties (PROMISE) G. D’Alessandro, University of Palermo 28206, Palermo, Italy; 2 Department of Neuroscience, Reproductive Sciences and Dentistry - Audiology Section, University of Naples Federico II 9307, Naples, Italy; 3 Urology Unit, University Hospital Policlinic, P. Giaccone 196020 , Palermo, Italy; 4 Microbiology and Virology Unit, University Hospital Policlinic, P. Giaccone 196020 , Palermo, Italy; 5 Internal Medicine Unit, University Hospital Policlinic, P. Giaccone 196020 , Palermo, Italy; University of Siena, Siena, Italy

**Keywords:** human papillomavirus, HPV, male, urine, genital swab, HPV non-invasive diagnosis

## Abstract

Human papillomavirus (HPV) represents the most prevalent sexually transmitted infection worldwide, often presenting as asymptomatic or subclinical in males. There is still uncertainty about the most suitable biological specimen for an accurate HPV diagnosis in males since the sampling site influences the performance of the diagnosis. Conventional diagnostic strategies rely on uncomfortable specimens for patients, such as urethral swabs. This study aimed to evaluate the diagnostic performance of using non-invasive, self-collected urine samples as an alternative to urethral swabs for detecting HPV infections in men. A total of 110 men provided a genital swab, a urethral swab, and a urine sample for analysis. HPV DNA was detected in 74.5% of the total samples, with 89.0% of HPV-positive samples harboring at least one oncogenic genotype. The most frequently detected genotypes were HPV53, HPV66, and HPV16. Of the individual specimens examined, genital swabs demonstrated the highest HPV positivity rate (68.2%), significantly outperforming urethral swabs and urine samples (*P* < 0.05). No significant difference was observed between urethral swabs and urine samples. However, the urine specimen alone demonstrated high specificity (100%), but limited sensitivity (53.7%), and moderate accuracy (65.5%). Receiver operating characteristic (ROC) analysis revealed that combining genital swabs with urine samples achieved excellent diagnostic performance (area under the ROC curve/AUC = 0.994), raising sensitivity to 98.8%, specificity to 100%, and accuracy to 99.1%. Overall, the results suggest that the combination of genital swabs and urine samples represents a reliable, non-invasive, and patient-acceptable alternative to conventional urethral sampling for HPV detection in men.

**IMPORTANCE:**

This study is important because it addresses a major unmet need in the diagnosis of HPV infection in men, namely, the lack of a standardized, reliable, and well-tolerated biological specimen. Using urine as a diagnostic sample, particularly in conjunction with genital swabs, has the potential to improve diagnostic efficacy while minimizing patient discomfort. A more acceptable diagnostic approach could enhance patient compliance, enable earlier detection of HPV infection, and improve clinical management. Furthermore, standardizing sampling strategies in men would help to close a critical gap in HPV prevention and control, with potential benefits for public health and reducing viral transmission.

## INTRODUCTION

Human papillomavirus (HPV) is recognized as the most common sexually transmitted infection (STI) worldwide, as the virus can spread through sexual activity but also non-penetrative contact (skin-to-skin contact) (1–3). Consequently, both men and women, if sexually active, can contract at least one time HPV infection during their lives (4, 5). So far, over 200 HPV genotypes have been identified, which are categorized by the International Agency for Research on Cancer (IARC) as high-risk (hrHPV) or low-risk (lrHPV) depending on their association with the development of cancerous lesions or genital warts, condylomas, respectively (6–8).

Persistent infection with a high-risk human papillomavirus (hrHPV) genotype, typically defined as lasting more than 12–24 months, significantly elevates the risk of developing precancerous lesions that harbor the potential to progress to invasive malignancy if appropriate treatment is withheld (9, 10).

HPV infection has been particularly closely associated with cervical cancer, which is the fourth most commonly diagnosed cancer in women (11–13). Indeed, available literature has focused heavily on HPV infection in women, neglecting the evaluation of infection in men. Furthermore, due to its proven association with cervical cancer, screening and prevention strategies for women are well developed, unlike those for men, as well as data on HPV infection acquisition (12, 14, 15).

Notwithstanding, the cause-effect relationship between HPV and the onset of anogenital cancers in males, such as anal and penile, is still significant, although less so, compared to cervical cancer (16–19).

Furthermore, it has been shown that men can act as reservoirs and vectors of the viral infection (20). Therefore, controlling and evaluating the infection in men could be relevant not only for preventing HPV-related malignancies in men but also for indirectly preventing cervical cancer in women (15, 21).

The most effective diagnostic approach for men appears to be the search for viral DNA since the infection is often asymptomatic or subclinical (20, 22, 23).

Currently, a significant knowledge gap remains regarding which sample is best suited to accurately diagnose male patients. The anatomical sites sampled, and the molecular tests used can affect the variability of the results (15).

Conventional methods for HPV DNA evaluation in men rely on collected swabs from the penile shaft, glans penis/coronal sulcus, scrotal, anal, or perianal region (23).

A previous study (22) comparing seminal fluid, genital swab, and urethral swab revealed that combining the latter two was the most effective diagnostic approach. However, some specimens, such as urethral swabs, are not well accepted by men themselves and can often be uncomfortable for patients (24).

The use of non-invasive samples, such as self-collected urine, as an alternative to urethral specimens may prove advantageous for the diagnosis of HPV infection. Due to their ease of collection, urine samples have already been proposed for diagnostic purposes in women, although less frequently in men, but some serious challenges remain to be addressed (13, 24–27).

In response to the clinical demand for non-invasive diagnosis, the present study aimed to evaluate the diagnostic performance of urine samples using a three-step concentration protocol as an alternative to the urethral swab conventionally used to identify male HPV infections, trying to achieve comparable diagnostic yield for the detection of HPV in the male population.

## MATERIALS AND METHODS

### Study population and sample collection

One hundred ten male patients aged 19 years or older who presented HPV-related symptoms or had an HPV-positive female partner were recruited for HPV testing at the Microbiology and Virology Laboratory of the Polyclinic “P. Giaccone” Hospital, Palermo, Department of Health Promotion, Mother and Child Care, Internal Medicine and Medical Specialties (PROMISE). This study does not aim to support population-based screening; rather, it aims to provide a tool for monitoring symptomatic or at-risk patients.

Three samples were performed, genital and urethral swabs, collected by the urologists of the Urology of Polyclinic “P. Giaccone” Hospital following a urological examination, and a first-void urine sample. The genital and urethral swabs (SIGMA TRANSWAB system ∑-Transwab; Medical Wire & Equipment Co. Bath Ltd.) were collected as described elsewhere (21).

All patients were instructed to wash their genital area with water only on the morning of the examination and to collect a urine sample from the first void, ensuring a volume of at least 50 mL was reached.

### Samples processing and DNA extraction

The urine samples were centrifuged at 2,000 rpm for 10 min. The resulting cellular pellet was then carefully resuspended in 1 mL of the original urine sample and subjected to a second, higher-speed centrifugation at 13,500 rpm for 5 min. After discarding the supernatant, the new pellet was resuspended in 1 mL of PBS (EuroClone S.p.A., Milan, Italy) to eliminate substances inhibiting DNA detection and then spun down at 13,500 rpm for 5 min. The resulting supernatant was discarded, and the pellet was either stored at −20°C or processed immediately.

Instead, the genital and urethral swabs were centrifuged at 13,000 rpm for 5 min to discard the supernatant and retain the pellet, which was then stored at −20°C or processed immediately.

The DNA extraction was performed by resuspending the pellet in 200 µL PBS using the automated extractor ELITe InGenius automated extractor through the magnetic bead technology of ELITe InGenius SP200 extraction reagent (Elitechgroup, Turin, Italy).

### HPV detection and genotyping

HPV-DNA detection was carried out using the CE-IVD INNO-LiPA HPV Genotyping Extra II diagnostic kit (Fujirebio, Tokyo, Japan), which can identify 32 different HPV genotypes, 20 hrHPV (HPV16, HPV18, HPV26, HPV31, HPV33, HPV35, HPV39, HPV45, HPV51, HPV52, HPV53, HPV56, HPV58, HPV59, HPV67, HPV68, HPV66, HPV70, HPV73, and HPV82) and 12 lrHPV (HPV6, HPV11, HPV40, HPV42, HPV43, HPV44, HPV54, HPV61, HPV62, HPV81, HPV83, and HPV89) based on the classification of the International Agency for Research on Cancer (IARC).

Samples that tested positive only for the HPV control probes and for which the genotype could not be identified were amplified using a two-step nested PCR with PGMY09/PGMY11 and GP05+/GP06+ primers, as previously described (28).

The resulting amplicons were then genotyped using Sanger sequencing on an ABI Prism 3130 Genetic Analyzer (Thermo Fisher Scientific, Waltham, MA), and sequence alignments were performed using NCBI’s Basic Local Alignment Search Tool (BLAST).

### Sample size

To evaluate the efficacy of the correct diagnosis of the combination of genital and urine samples in detecting male HPV infection, we estimated the minimum sample size statistically significant for patients in this study.

The sample size was defined using Cochran’s formula. Particularly, we considered a *z*-score at 99%, an error *e* = 10%, and a prevalence of correct diagnosis *P* = 43% according to E. Lazcano-Ponce et al. (29) which considered the combination of genital and urethral swabs on 114 subjects.

Therefore, the correct diagnosis for detecting males infected with HPV, as determined by a combination of genital swabs and urine samples, was estimated within this interval [33%; 53%].

Based on the previous consideration, the minimum estimated sample size is 94 patients. Finally, to reduce statistical biases due to information/data loss caused by unexpected events, the sample size is enlarged to 110 patients.

### Statistical analysis

Data were presented as number and percentage for categorical variables, and continuous data were expressed as the mean and standard deviation (SD) or median and interquartile range (IQR = [*Q*1, *Q*3]).

Cochran’s *Q* test was performed to evaluate significant differences in proportions or percentages between three or more matched sets of frequencies or proportions. If the Cochran’s *Q* test is positive (*P* < 0.05), the minimum required difference for a significant difference between two proportions was calculated (30).

Chi-square test and Fisher’s exact test were performed to evaluate significant differences in proportions or percentages between the two groups. The multiple comparison chi-square and Fisher’s exact test were used to define significant differences among three or more percentages for unpaired data. If the chi-square or Fisher’s exact test were significant (*P* value < 0.05), the post hoc test was performed using the Adjusted Standardized Residuals and the *Z*-test. Fisher’s exact test was used where the chi-square test was not appropriate. To compare three or more modalities of a variable, the chi-square goodness of fit was used.

The test for normal distribution was performed using the Shapiro-Wilk test. The *t*-test was used to evaluate the differences between two means of unpaired or paired data. Alternatively, the Mann-Whitney test or Wilcoxon test were, respectively, used if the data distributions were not normal.

Additionally, to evaluate the performance of genital swabs, urethral swabs, and urine samples in the detection of HPV infection, the sensitivity, specificity, accuracy, and Receiver Operating Characteristic (ROC) curve were computed considering gold diagnostic the variable total. The total variable was a dichotomized variable represented by Boolean sum of the results (positive and negative to HVP) of genital swabs, urethral swabs, and urine samples. Particularly, for pairwise comparison of areas under ROC curves (AUCs), the DeLong test was used.

Given the lack of an independent gold standard for the diagnosis of HPV infection in males, a composite reference outcome (Total) was defined using currently accepted sampling methods, including genital swabs, urethral swabs, and urine samples. A subject was classified as HPV-positive if at least one of the collected specimens tested positive. Using this composite reference outcome, diagnostic accuracy analyses were performed for both individual sampling methods and paired combinations, with the aim of identifying less invasive strategies whose sensitivity and overall performance most closely approximated that obtained when all three sampling sites were considered together.

While the diagnostic reference outcome (Total) included the index tests themselves, false-positive results could not occur by definition. As a consequence, estimates of specificity and negative predictive value (NPV) were inherently inflated and do not reflect true diagnostic accuracy. For this reason, specificity and NPV were reported for descriptive purposes only, while sensitivity and relative performance were the primary focus of the analysis. This limitation applies to both single and paired sampling strategies, as all contributed to the construction of the composite reference outcome.

Finally, all tests with *P* value < 0.05 were considered significant. The statistical analysis was performed using the Matrix Laboratory (MATLAB) analytical toolbox version 2008 (MathWorks, Natick, MA, USA). For Windows at 32 bits.

## RESULTS

The cohort consisted of 110 men, aged between 19 and 76 years, with an average age of 41.2 years (standard deviation: 12.0 years). The main reason for testing was having had a female partner who evaluated positive for HPV, reported by 74.5% (82/110).

Table 1 presents the patients' general characteristics.

**TABLE 1:** General characteristics and lifestyle habits of the study cohort (110 males)

Parameters	Sample data
Patients (males)	110
Age	
Mean (SD)	41.2 ± 12.0
Median (IQR)	38.0 (33.0, 50.0)
Symptom	
No symptom	71.8% (79)
Condylomas	28.2% (31)
Smoke	
No	41.8% (46)
Ex	10.9% (12)
<10/DIE	5.5% (6)
≥10/DIE	41.8% (46)
Alcohol	
No	32.7% (36)
Rarely	55.5% (61)
Frequently	11.8% (13)
Lifetime number of sexual partners	
[1, 10]	35.4% (39)
[10, 20]	36.4% (40)
[20, 30]	10.0% (11)
[30, 40]	5.5% (6)
[40, 50]	1.8% (2)
≥50	10.9% (12)
Age at first sexual intercourse	
Mean (SD)	17.4 (2.7)
Median (IRQ)	17.0 (16.0, 18.0)
Years with a steady partner	
Mean (SD)	6.8 (9.4)
Median (IRQ)	2.0 (1.0, 10.0)
Condom use	
No	41.8% (46)
Yes	58.2% (64)
Education level	
Lower secondary school	17.3% (19)
High school	46.4% (51)
University	31.8% (35)
Postgraduate	4.5% (5)
Vaccination	
No	87.3% (96)
Yes	12.7% (14)
Motivation at first admission	
Partner positive to HPV	74.5% (82)
Risky behavior	7.3% (8)
Lesions	18.2% (20)

Of the samples analyzed, 74.5% (82/110) were positive for human papillomavirus. Within the HPV-positive cohort, multiple infections were the predominant pattern (69.5%, 57/82) compared to single-genotype infections (30.5%, 25/82). Most positive samples (89.0%, 73/82) harbored at least one oncogenic HPV type. Only 11.0% (9/82) displayed exclusively low-risk HPV genotypes (lrHPVs).

Table 2 reports the results for the total samples and for the genital swab (GS), the urethral swab (US), and the urine sample (U).

**TABLE 2:** HPV positivity for the study cohort and for each type of sample (genital swab, urethral swab, and urine sample)

Parameters	Total	Genital swabs	Urethral swabs	Urine samples
Patients positive to HPV	74.5% (82/110)	68.2% (75/110)	49.1% (54/110)	40.0% (44/110)
Risk group				
hr/lrHPV	89.0% (73)	85.3% (64)	85.2% (46)	81.8% (36)
lrHPV	11.0% (9)	14.7% (11)	14.8% (8)	18.2% (8)
Nr. of genotypes				
One	30.5% (25)	36.0% (27)	31.5% (17)	45.5% (20)
Two	18.3% (15)	20.0% (15)	27.8% (15)	22.7% (10)
Three	20.7% (17)	21.3% (16)	18.5% (10)	15.9% (7)
Four	11.0% (9)	12.0% (9)	16.7% (9)	9.1% (4)
Five	11.0% (9)	8.0% (6)	3.7% (2)	2.3% (1)
Six	7.3% (6)	1.3% (1)	0.0% (0)	4.5% (2)
Seven	0.0% (0)	0.0% (0)	1.9% (1)	0.0% (0)
Eight	1.2% (1)	1.3% (1)	0.0% (0)	0.0% (0)

Of the 68.2% (75/110) of the HPV-positive genital swabs identified, 64% (48/75) showed multiple infections and 36% (27/75) single infections. Specifically, hrHPVs, either alone or alongside lrHPVs, were detected in 85.3% (64/75) of cases, whereas lrHPVs were present in only 14.7% (11/75) of cases.

In contrast, the positive urethral swabs were 49.1% (54/110), showing multiple and single infections in 68.5% (37/54) and 31.5% (17/54), respectively. hrHPV genotypes were found in 85.2% (46/54) of cases and lrHPV in 14.8% (8/54).

Similarly, regarding urine samples, 40.0% (44/110) were HPV-positive, of which 54.5% (24/44) showed multiple infections and 45.5% (20/44) single infections. Oncogenic HPV genotypes were shown in 81.8% (36/44) and non-oncogenic genotypes in 18.2% (8/44).

Additionally, we found significant differences among genital swabs, urethral swabs, and urine samples (*P* < 0.001, Cochran’s *Q* test). Particularly, by *post hoc* test, a significant difference between genital swabs and urethral swabs (68.2% vs 49.1%, *P* < 0.05) and between genital swabs and urine samples (68.2% vs 40.0%, *P* < 0.05) was found. Finally, no significant difference was observed between urethral swabs and urine samples (49.1% vs 40.0%, *P* > 0.05).

Table 3 shows the genotypes detected in the study cohort and in each individual sample of the 82 HPV-positive patients.

**TABLE 3:** Distribution and frequency of HPV genotypes among HPV-positive patients according to sampling site

Genotype	Total	Genital swabs	Urethral swabs	Urine samples
6	11.0% (9)	12.0% (9)	9.3% (5)	6.8% (3)
11	2.4% (2)	2.7% (2)	1.9% (1)	2.3% (1)
16	15.9% (13)^*a*^	14.7% (11)^*a*^	11.1% (6)	15.9% (7)^*a*^
18	6.1% (5)	6.7% (5)	3.7% (2)	4.5% (2)
31	12.2% (10)	8.0% (6)	11.1% (6)	9.1% (4)
33	2.4% (2)	2.7% (2)	0.0% (0)	0.0% (0)
35	6.1% (5)	4.0% (3)	9.3% (5)	9.1% (4)
39	6.1% (5)	6.7% (5)	1.9% (1)	6.8% (3)
40	3.7% (3)	2.7% (2)	1.9% (1)	2.3% (1)
42	13.4% (11)	13.3% (10)	9.3% (5)	11.4% (5)
43	6.1% (5)	5.3% (4)	5.6% (3)	4.5% (2)
44	8.5% (7)	9.3% (7)	1.9% (1)	4.5% (2)
45	8.5% (7)	6.7% (5)	7.4% (4)	2.3% (1)
51	13.4% (11)	13.3% (10)	11.1% (6)	9.1% (4)
52	13.4% (11)	12.0% (9)	7.4% (4)	6.8% (3)
53	25.6% (21)^*a*^	22.7% (17)^*a*^	22.2% (12)^*a*^	15.9% (7)^*a*^
54	7.3% (6)	6.7% (5)	3.7% (2)	2.3% (1)
56	11.0% (9)	6.7% (5)	11.1% (6)	6.8% (3)
58	11.0% (9)	9.3% (7)	9.3% (5)	13.6% (6)
59	12.2% (10)	8.0% (6)	14.8% (8)^*a*^	13.6% (6)
61	11.0% (9)	10.7% (8)	13.0% (7)	15.9% (7)^*a*^
62	3.7% (3)	4.0% (3)	3.7% (2)	2.3% (1)
66	18.3% (15)^*a*^	16.0% (12)^*a*^	20.4% (11)^*a*^	13.6% (6)
67	6.1% (5)	2.7% (2)	5.6% (3)	4.5% (2)
68	9.8% (8)	6.7% (5)	5.6% (3)	4.5% (2)
70	6.1% (5)	5.3% (4)	7.4% (4)	4.5% (2)
73	12.2% (10)	12.0% (9)	9.3% (5)	9.1% (4)
81	1.2% (1)	1.3% (1)	1.9% (1)	2.3% (1)
82	2.4% (2)	2.7% (2)	1.9% (1)	0.0% (0)
83	1.2% (1)	0.0% (0)	1.9% (1)	2.3% (1)
84	1.2% (1)	1.3% (1)	1.9% (1)	2.3% (1)
87	1.2% (1)	0.0% (0)	1.9% (1)	0.0% (0)
89	11.0% (9)	8.0% (6)	13.0% (7)	4.5% (2)
Total	231	185	130	94

^*a*^ More significant frequent genotype.

The predominant and statistically significant human papillomavirus genotypes detected across the entire study cohort were HPV53 (25.6%, *P* < 0.0001), HPV66 (18.3%, *P* = 0.0017), and HPV16 (15.9%, *P* = 0.0193). Notably, all of these most frequently observed genotypes were classified as high-risk HPV (hrHPV) types.

Regarding the genital swabs, the most frequently detected significant HPV genotypes were HPV53 (22.7%, *P* < 0.0001), HPV66 (16.0%, *P* =0.0166), and HPV16 (14.7%, *P* = 0.0193). We also observed genital swabs that the most frequent genotypes were all hrHPV genotypes.

In the urethral swabs, the most frequently detected significant HPV genotypes were HPV53 (22.2%, *P* < 0.0001), HPV66 (20.4%, *P* = 0.0166), and HPV59 (14.8%, *P* = 0.0193). We also observed that the most frequent genotypes were all hrHPV genotypes.

Finally, in urine samples, the most detected significant genotypes were HPV53 (15.9%, *P* = 0.0102) and HPV16 (15.9%, *P* = 0.0102), whereas the most detected lrHPV was HPV61 (15.9%, *P* = 0.0102). In contrast to the swab sites, in the urine samples, the most frequent genotypes include hr and lrHPVs.

Table 4 shows the concordance rates observed among the different combinations of the three samples Specifically, positive concordance (all three samples tested positive) was achieved in 30% (33/110). Conversely, negative concordance (all three samples tested negative) was observed in 25.45% (28/110) of the cohort.

**TABLE 4:** Frequency of result combinations across genital swab, urethral swab, and urine samples

Urethral swabs	Genital swabs		Urine samples
	Positive	Negative	
Positive	33	4	Positive
Positive	16	1	Negative
Negative	5	2	Positive
Negative	21	28	Negative

**Fig 1:**
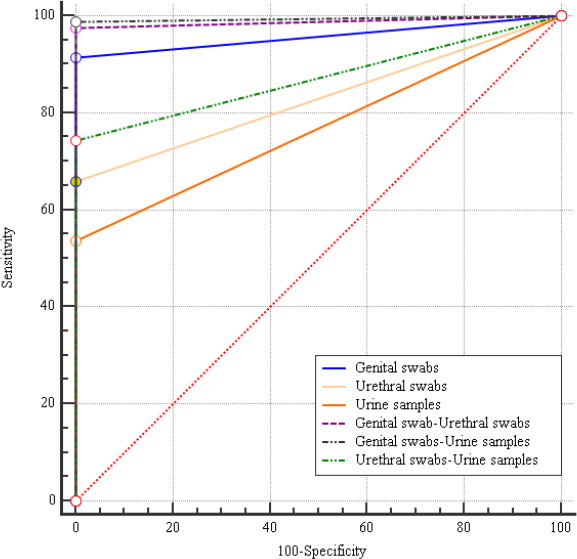
Comparison of area under the ROC curves among GS, US, U, GS-US, GS-U, and US-U.

To evaluate the best diagnostic performance among genital swabs, urethral swabs, and urine samples, we computed the sensitivity, specificity, accuracy (Table 5), and Receiver Operating Characteristic (ROC) curve, considering gold diagnostic the variable Total (Fig. 1 and Table 6).

**TABLE 5:** Diagnostic performance of paired sampling strategies (genital swab–urethral swab, genital swab–urine sample, and urethral swab–urine sample) for HPV detection

Sample	Sensitivity (CI at 95%)	Specificity (CI at 95%)	Accuracy (CI at 95%)
Genital swabs (GS)	91.5% (84.2%, 95.9%)	100% (95.8%, 100%)	93.6% (86.9%, 97.5%)
Urethral swabs (US)	65.9% (56.1%, 74.5%	100% (95.8%, 100%)	74.6% (65.2%, 82.3%)
Urine samples (U)	53.7% (43.9%, 63.1%)	100% (95.8%, 100%)	65.5% (55.7%, 74.2%)
GS-US	97.6% (92.1%, 99.9%)	100% (95.8%, 100%)	98.2% (92.9%, 100%)
GS-U	98.8% (93.8%, 100%)	100% (95.8%, 100%)	99.1% (94.3%, 100%)
US-U	74.4% (65.0%, 82.1%)	100% (95.8%, 100%)	80.9% (72.1%, 87.7%)

**TABLE 6:** Pairwise comparison of area under the ROC curves^*a*^

AUC (*P* value)	Genital swabs	Urethral swabs	Urine samples	GS-US	GS-U	US-U
Genital swabs		0.957 vs 0.829 *P* < 0.0001*	0.957 vs 0.768 *P* < 0.0001*	0.957 vs 0.988 *P* = 0.0218*	0.957 vs 0.994 *P* = 0.0114*	0.957 vs 0.872 *P* = 0.0060*
Urethral swabs	0.829 vs 0.957 *P* < 0.0001*		0.829 vs 0.768 *P* = 0.0373*	0.829 vs 0.988 *P* < 0.0001*	0.829 vs 0.994 *P* < 0.0001*	0.829 vs 0.872 *P* = 0.0060*
Urine samples	0.768 vs 0.957 *P* < 0.0001*	0.768 vs 0.829 *P* = 0.0373*		0.768 vs 0.988 *P* < 0.0001*	0.768 vs 0.994 *P* < 0.0001*	0.768 vs 0.872 *P* < 0.0001*
GS-US	0.988 vs 0.957 *P* = 0.0218*	0.988 vs 0.829 *P* < 0.0001*	0.988 vs 0.768 *P* < 0.0001*		0.988 vs 0.994 *P* = 0.565	0.988 vs 0.872 *P* < 0.0001*
GS-U	0.994 vs 0.957 *P* = 0.0114*	0.994 vs 0.829 *P* < 0.0001*	0.994 vs 0.768 *P* < 0.0001*	0.994 vs 0.988 *P* = 0.565		0.994 vs 0.872 *P* < 0.0001*
US-U	0.872 vs 0.957 *P* = 0.0060*	0.872 vs 0.829 *P* = 0.0060*	0.872 vs 0.768 *P* < 0.0001*	0.872 vs 0.988 *P* < 0.0001*	0.872 vs 0.994 *P* < 0.0001*	

^*a*^ GS-US, genital swabs with urethral swabs; GS-U, genital swabs with urine samples; US-U, urethral swabs and urine samples; *, significant test.

In Table 5, we reported the sensitivity, specificity, and accuracy for genital swabs, urethral swabs, and urine samples diagnostic referred to total variable diagnostic (genital swabs, urethral swabs, and urine samples). Additionally, sensitivity, specificity, and accuracy were also computed for the combination of genital swabs with urethral swabs (GS-US), of genital swabs with urine samples (GS-U), and urethral swabs with urine samples (US-U).

In Fig. 1, we reported the comparison of area under the ROC curves among genital swabs, urethral swabs, urine samples, genital-urethral swabs, genital swab-urine, and urethral swab-urine.

Fig. 1 shows the area under the ROC curve (AUC) for genital swabs, urethral swabs, urine samples, genital swabs-urethral swabs, genital swabs-urine samples, and urethral swabs-urine samples, referred to results obtained by the total variable, i.e., by combination of genital swabs, urethral swabs, and urine samples. In Table 6, we reported the details of the pairwise comparison of ROC curves.

In Table 6, we found the best-performing diagnostic samples were GS-US (AUC: 0.988) and GS-U (AUC: 0.994) and an accuracy equal to 98.2% and 99.1%, respectively. Additionally, GS-U showed an AUC greater than GS-US but not significant (0.994 vs 0.988, *P* = 0.565), i.e., GS-U and GS-US had statistically equal performance.

## DISCUSSION

To date, research into human papillomavirus (HPV) diagnosis has focused predominantly on women, driven by the critical need to reduce cervical cancer rates (1, 17, 21, 31). However, in a recent study, we demonstrated that HPV infection in male partners is associated with both the incidence and severity of cervical lesions in women (21).

The incidence of anogenital cancers in men is increasing at an alarming rate, representing a growing public health crisis. Furthermore, men have long been recognized as silent and often asymptomatic reservoirs of the virus. As a result, they may unknowingly contribute to viral transmission, while lower participation in diagnostic testing can further facilitate the persistence and spread of the infection (16–20, 24).

Men are particularly reluctant to undergo tests that require penile swabs, especially urethral swabs, as these are perceived as invasive (23, 24, 32). The literature addressing the optimal specimen type for HPV detection in males is somewhat dated; nonetheless, different studies about the male population have assessed the diagnostic performance of various sample types, both individually and in comparative analyses (22, 23, 25, 26, 29, 33–36). Despite this, no definitive gold-standard specimen has been established (25, 32). In particular, the use of urine for HPV detection remains a subject of ongoing debate.

The objective of this study was to evaluate the diagnostic performance of urine sampling as a noninvasive alternative to the traditional urethral swab for HPV detection. By analyzing urine as a noninvasive specimen, we aimed to overcome significant barriers to patient compliance with male HPV follow-up, providing a more acceptable and easily repeatable approach for longitudinal monitoring. This strategy could encourage adherence to clinical surveillance protocols, particularly among individuals who are symptomatic or at risk, by minimizing discomfort and making repeated sampling over time more practical.

The study involved a cohort of 110 male participants, each of whom provided a genital swab, a urethral swab, and a urine sample for comparative analysis

The male cohort demonstrated an overall HPV-positivity rate of 74.5%, determined by the detection of the virus in at least one of the collected specimens (Table 2). Comparable prevalence estimates have been reported in previous studies. Giuliano and colleagues (23), assessing multiple anatomical sites, observed HPV positivity at one or more locations in 64.5% of participants. Similarly, Giovannelli et al. (22), who tested genital swabs, urine, and semen samples, reported an overall positivity rate of 72%, and Koene et al. (36), who evaluated urine sample using penile swab as gold standard specimen, showed an overall HPV prevalence of 75%.

Of the three specimens, the genital swab yielded the highest HPV positivity rate (68.2%) in our cohort, showing significant differences compared to the urethral swab and urine samples (*P* < 0.05). Similar findings regarding the effectiveness of genital swabs have been reported by Giuliano et al. (23), Giovannelli et al. (22), Weaver et al. (33), and Bruni et al. (5).

Nonetheless, no significant difference was observed between urethral swabs and urine samples, indicating the limited utility of the urethral swab; a sample that is, moreover, generally not preferred by patients. This finding aligns with previous studies, such as those by Giuliano et al. (23), Lazcano-Ponce et al. (29), Giovannelli et al. (22), and Weaver et al. (33), which consistently showed lower HPV detection rates in urethral swabs and urine compared with genital specimens. However, none of these investigations employed a three-step urine concentration protocol, as implemented in the present study.

Bober et al. (37) provided recommendations to optimize HPV DNA detection in urine samples from women, which could also be applied to men with certain adaptations. Our study cohort also received the following effective advice: use first-void urine, collect sufficient volume for subsequent concentration, use a targeted diagnostic test for the desired purpose, and avoid cleaning the genital area before collecting the sample.

However, adherence to these instructions could not be objectively verified, and it cannot, therefore, be excluded that variability in patient compliance may have influenced the sensitivity of urine-based HPV detection in our cohort.

The most prevalent genotypes detected in our study cohort were HPV53 (25.6%, *P* < 0.0001), HPV66 (18.3%, *P* = 0.0017), and HPV16 (15.9%, *P* = 0.0193) (Table 3). These findings are consistent with our previous research in the male population from the same region (21, 38), which similarly identified HPV53 and HPV66 as the most frequent genotypes.

Besides, certain specimen types appear to be more proficient at detecting specific HPV genotypes. Urine samples and genital swabs, for instance, seem particularly efficient in identifying HPV16 infections, consistent with the findings of Pathak et al. (39), whose meta-analysis reported high specificity of these specimens for the most known oncogenic genotypes, HPV16 and HPV18, in women.

In contrast, Delgado López et al. (32) observed that genotypes such as HPV16, 18, 39, 52, and 66 were detected exclusively in semen when comparing urine and semen samples. On the other hand, Aung et al. (40) reported that urine samples were more effective in detecting low-risk HPVs, such as HPV6 and HPV11. In this regard, in our study, the urine sample was also more effective at detecting HPV61 (15.9%, *P* = 0.0102) than other specimen types.

Despite these variations, an overall agreement exceeding 50% was observed (comprising 30% positive and 22.45% negative agreement), suggesting a satisfactory diagnostic yield across the three sample types (Table 4).

Conversely, urine samples demonstrated high diagnostic specificity (100%), albeit with relatively low sensitivity and overall accuracy (53.7% and 65.5%, respectively) (Table 5). These results are comparable to those reported by Koene et al. (36) and Delgado-López et al. (32), who found lower specificity and sensitivity percentages. The former study found specificity and sensitivity percentages of 95% and 41%, respectively, while the latter study found percentages of 95.6% and 37.5%. These results suggest that a urine sample alone may not be the most effective specimen for detecting HPV infection.

The previous point is supported by the analysis of the area under the ROC curve (AUC), which indicated that a combined approach using genital swabs and urine samples could be the most effective diagnostic method, given the high levels of sensitivity (98.8%), specificity (100%), and accuracy (99.1%) observed.

Indeed, the best-performing diagnostic samples seem to be the combination of a genital swab and a urethral swab (AUC: 0.988) or a genital swab and a urine sample (AUC: 0.994) (Table 6). Our analysis shows that the two combinations perform equally statistically.

Although the two combinations perform similarly, the non-invasive nature and straightforward process of providing a urine sample could be preferred by men.

The strategy of combining another sample, such as a genital swab, with the urine specimen for an accurate diagnosis appears to improve diagnostic efficiency for two reasons. First, for the well-established diagnostic performance of the genital swab, already described in literature (5, 22, 23, 33). Second, because a concentration protocol could improve urine efficiency. Other studies have suggested that urine concentrations are an effective means of detecting HPV-DNA (25, 26, 32, 36). Nevertheless, none of them proposed a protocol for ultra-concentrating urine and removing potential inhibitors. We would like to emphasize that future studies directly comparing low-first-void urine with larger-volume samples, and systematically evaluating different processing and pre-analytical protocols, are needed to determine the most effective, reproducible strategy for detecting HPV in men. It is worth noting in this context that our study is one of the few (32, 41) to have employed a relatively high urine volume (50 mL) and a more elaborately executed sample processing, which could affect samples with low viral load. Future investigations should focus on establishing a single standardized protocol by systematically comparing the various procedures already proposed in the literature.

Notwithstanding, the combination with the genital swab supports the prospect and feasibility of using urine samples for diagnosing HPV infection in the future.

Overall, these findings demonstrate that urine samples, when used alongside genital swabs, are a clinically applicable alternative to conventional urethral specimens, offering comparable sensitivity and diagnostic yield. This combined approach may address the growing clinical demand for noninvasive diagnostic strategies, with the potential to improve patient compliance while maintaining good performance. However, it is not currently intended for implementation in population-level screening programs and may be better suited for evaluating and longitudinally monitoring symptomatic or at-risk individuals in clinical settings. Further studies are nevertheless required to validate these findings and establish standardized protocols to ensure reproducible results.

### Future perspectives

Our study suggests that certain sample types may exhibit greater sensitivity and efficiency in detecting specific HPV genotypes, indicating that the choice of biological matrix can influence genotype-specific detection rates. However, the analysis was limited to mucosal genotypes, leaving cutaneous HPV types unexplored and thereby providing only a partial view of the overall HPV diversity. Building on these findings, an important direction for future research could be to assess whether the prevalence and distribution of cutaneous HPV genotypes differ across anatomical sites (e.g., the genital area, the urethra, and urine samples). Furthermore, it is worth noting that Karimi et al. (42) have already reported site-specific differences in HPV prevalence, highlighting distinct patterns within the oral cavity in the context of head and neck cancers, thereby supporting the hypothesis of anatomical niche-dependent variability.

### Limitations

This study has some limitations that should be considered when interpreting the results. First, an independent gold standard for the diagnosis of HPV infection in men is currently lacking. As a consequence, no single sampling method can be considered definitively representative of true infection status. In this context, the combined evaluation of genital swab, urethral swab, and urine sample was used to represent the most comprehensive assessment of HPV detection achievable using currently accepted diagnostic approaches.

Because this composite assessment is derived from the same sampling methods evaluated in the study, it should not be interpreted as an absolute reference standard. The aim of the study was not to validate a novel diagnostic test, but rather to compare different single and combined sampling strategies and to identify the sampling methods or pairs of samples that most closely approximate the overall detection achieved when all three sampling sites are considered together.

Importantly, when combinations of two sampling methods are evaluated, negative results for a given pair may coexist with a positive result in the third sampling site. This allows meaningful comparison among sampling combinations and supports the use of comparative diagnostic performance measures. The diagnostic accuracy metrics reported in this study should be interpreted in a relative and comparative manner, focusing on the ability of different sampling strategies to reproduce the most comprehensive diagnostic assessment currently available, rather than as measures of absolute diagnostic validity.

## Data Availability

The data supporting the findings of this study are available from the corresponding author on request and are subject to institutional and ethical restrictions.
